# Multifunctional Waterborne Polyurethane Microreactor-Based Approach to Fluorocarbon Composite Latex Coatings with Double Self-Healing and Excellent Synergistic Performances

**DOI:** 10.3390/nano12234216

**Published:** 2022-11-27

**Authors:** Chao Li, Huimin Guo, Ning Zhang, Yao Jin, Kai Han, Jinfeng Yuan, Zhicheng Pan, Mingwang Pan

**Affiliations:** 1 Department of Polymer Materials and Engineering, School of Chemical Engineering and Technology, Hebei University of Technology, Tianjin 300130, China; 2 Hebei Key Laboratory of Functional Polymers, Hebei University of Technology, Tianjin 300130, China

**Keywords:** double self-healing model, multifunctional waterborne polyurethane, soap-free emulsion polymerization

## Abstract

In this article, chlorotrifluoroethylene (CTFE)-based fluorocarbon composite latexes and their coatings are successfully fabricated by an environmentally friendly preparation method based on a new multifunctional waterborne polyurethane (MFWPU) dispersion. It is worth noting that the MFWPU acts as the sole system stabilizer as well as microreactor and simultaneously endows the composite coating with excellent double self-healing performance and adhesion. Moreover, the introduction of a dynamic disulfide bond in the polyurethane dispersion entrusts the coating with excellent scratch self-healing performance. Simultaneously, carbon–carbon double bonds in the polyurethane dispersion increase the compatibility between the core polymer and shell polymer. The fluorine-containing chain segments can be distributed in the coating evenly during the self-assembly film-forming process of composite particles so that the original element composition of the worn coating surface can restore the original element composition after heating, and the coating presents a regeneration ability, which further and verifies the usefulness of the double self-healing model of the coating. Afterward, efficient recovery and durability, which are two contradictory properties of scratch self-healing polymers, are optimized to obtain a composite coating with excellent comprehensive performance. The research results regarding the composite system may provide a valuable reference for the structural design and application of waterborne fluorocarbon functional coatings in the future.

## 1. Introduction

Waterborne coatings are prepared by replacing the diluent in traditional coatings with water. They are nonflammable, odorless and have low volatile organic compounds (VOCs) compared with traditional organic solvent coatings [1,2,3] Therefore, it is safer, healthier and more environmentally friendly to use waterborne coatings in technology [4,5,6] However, waterborne coatings still have some disadvantages at present, such as poor water resistance, weak weather resistance and so on [7,8] Accordingly, it is necessary to develop high-performance waterborne coatings and broaden their application fields. Recently, waterborne fluorocarbon coatings have been developed as waterborne coatings with broad prospects, the remarkable feature of which is that the molecular structure of the fluorocarbon resin, which contains a large number of fluorine–carbon bonds (F-C) so that the fluorine-containing segments are stable in the face of light, heat and chemical effects [9,10,11]. High electronegative and low polarizable fluorine atoms with a quite small van der Waals radius (1.32 Å) invest the F-C bond with a short bond length and high bond energy (485 kJ/mol) [12,13,14]. Therefore, the fluorocarbon resin exhibits excellent weather resistance, heat resistance and solvent resistance, and it can be widely used in ships, railways, bridges, separation film, fabric finishing, microelectronics, aerospace, household appliance, solar energy, cultural relic protection and other fields [15,16,17]. Among these fluoropolymer resins, polychlorotrifluoroethylene (PCTFE) has attracted the attention of the public owing to its outstanding weather resistance and chemical resistance. Therefore, it is an indispensable fluorine-containing polymer. However, PCTFE always presents a high crystallinity, resulting in a poor film-forming property and weak adhesion to the substrate. As a consequence, it is difficult to use it as an aqueous coating alone, and the manufacturing cost is high. Undoubtedly, the design and synthesis of CTFE copolymer have always been the focus of research in the field of fluorocarbon because the copolymer can lead to disorder in macromolecular structure and reduce the crystallinity [18,19,20]. Interestingly, isobutyl vinyl ether (IBVE) cannot undergo free radical homopolymerization, but it can react with CTFE to form alternating copolymers, which is caused by the electron acceptance of CTFE (e = +1.56) and an electron donation from IBVE (e ≈ −2) [21,22,23].

Although the copolymer of CTFE and IBVE has good film-forming properties [24,25], there are still several problems for the P(CTFE-*alt*-IBVE) fluorocarbon resin emulsion type in latex synthesis, film formation and coating properties. On the one hand, traditional small-molecule surfactant created in the synthesis process of fluorocarbon resin latex is difficult to remove completely because its large dosage causes a decrease in water resistance, antifouling performance, adhesion and coating strength, which limits the application of fluorocarbon resin latex. Oligomer surfactants are a novel type of surfactant, which possesses a series of unique properties, such as nontoxicity or low toxicity, easy synthesis and modification, and it can be endued with special functions. Among them, waterborne polyurethane oligomer surfactant has great practical value and development prospects due to its unique structure and designability, such as the adjustable type, size and position of hydrophilic and hydrophobic groups [16,26]. On the other hand, their adhesion to various substrates still needs to be greatly improved, despite the good film-forming properties of CTFE and IBVE copolymers [27,28]. In this way, a method of endowing the coating with excellent film-forming performance and adhesion while still maintaining the excellent characteristics of CTFE polymer has great significance. The coating will suffer more or less physical damage during use, such as impact scratches or surface friction and wear, resulting in scratches, fractures or damage to the chemical components on the coatings surface, which may reduce the performance and service life of the coating. Therefore, it is important to prepare the coatings with self-healing properties in order to prolong the service life and reduce the maintenance cost of coating applications [29,30,31]. Nevertheless, since the self-healing process requires a higher molecular movement ability, the currently reported intrinsic self-healing materials are mostly hydrogels or soft elastomers, and the research on their use in waterborne coatings is scant [32,33,34]. In addition, efficient recovery and durability (including thermal stability, water resistance and so on), which are regarded as the most important properties of scratch self-healing polymers, are difficult to optimize at the same time because both are often contradictory; as such, there is difficulty in achieving the two properties simultaneously in one material [35,36].

In the present work, a reactive polyurethane microreactor with a dynamic disulfide bond (S-S) named MFWPU was synthesized using phase inversion emulsification, and it was used to emulsify and stabilize the copolymerization of CTFE and IBVE monomers. Finally, a series of P(CTFE-*alt*-IBVE)/MFWPU composite latex particles were successfully prepared with soap-free emulsion polymerization. The use of MFWPU can not only solve the problems caused by traditional small molecule surfactant, film-forming and adhesion of CTFE coatings but also solve the scratch and wear problems of CTFE coatings at the same time. A coating scratch–friction double self-healing model was further proposed on the basis of investigating the effects of dynamic S-S and active carbon–carbon double bonds (C=C) in MFWPU on the scratch self-healing performance and surface element composition of composite coatings. In addition, the effects of P(CTFE-*alt*-IBVE)/MFWPU composite particles with different core–shell mass ratios on the scratch self-healing, thermal stability and water resistance of the coating were systematically studied to optimize the contradictory properties of efficient recovery and durability in the self-healing polymer. Finally, a P(CTFE-*alt*-IBVE)/MFWPU waterborne fluorocarbon coating with excellent film-forming, adhesion, weather resistance and scratch–friction double self-healing performance was obtained smoothly.

## 2. Experimental Section

### 2.1. Materials

Polytetrahydeofuran ether glycol (PTHF, *M*_n_ ≈ 1000) (Shanghai Aladdin Biochemical Technology Co., Ltd., Shanghai, China) and 2,2-dimethylpropionic acid (DMPA) (Shandong Wanduofu Chemical Reagent Co., Ltd., Zibo, China) were dehydrated in a vacuum at 110 °C or 120 °C for 2 h before use. N,N-dimethylformamide (DMF) and dibutyltin dilaurate (DBTDL) were provided by Tianjin Fuchen Chemical Reagent Co., Ltd. (Tianjin, China). Isophorone diisocyanate (IPDI) was supplied by Shanghai Heshibi Chemical Reagent Co., Ltd. (Shanghai, China). 2,2′-disulfide diethanol was purchased from AlfaAesar Chemical Co., Ltd. (Shanghai, China). Dimethylaminoethyl methacrylate (DMAEMA) (Shanghai Aladdin Biochemical Technology Co., Ltd., Shanghai, China) was distilled under reduced pressure (30 mmHg) at 68 °C. Chlorotrifluoroethylene (CTFE) was provided by Jiangsu Blue Planet Environmental Protection Technology Co., Ltd. (Changzhou, China). Isobutyl vinyl ether (IBVE), triethylamine (TEA) and 1,4-butanediol (BDO) were purchased from Shanghai Aladdin Biochemical Technology Co., Ltd. (Shanghai, China). Potassium persulfate (KPS, >99.5%) (Traditional Chinese Medicine Group Chemical Reagent Co., Ltd., Shanghai, China) and sodium bicarbonate (NaHCO_3_; Tianjin Fuchen Chemical Reagent Co., Ltd., Tianjin, China) were used as the initiator and buffer, respectively.

### 2.2. Polymerization Procedure

#### 2.2.1. Synthesis of Multifunctional Waterborne Polyurethane Dispersion (MFWPU)

**Figure 1:**
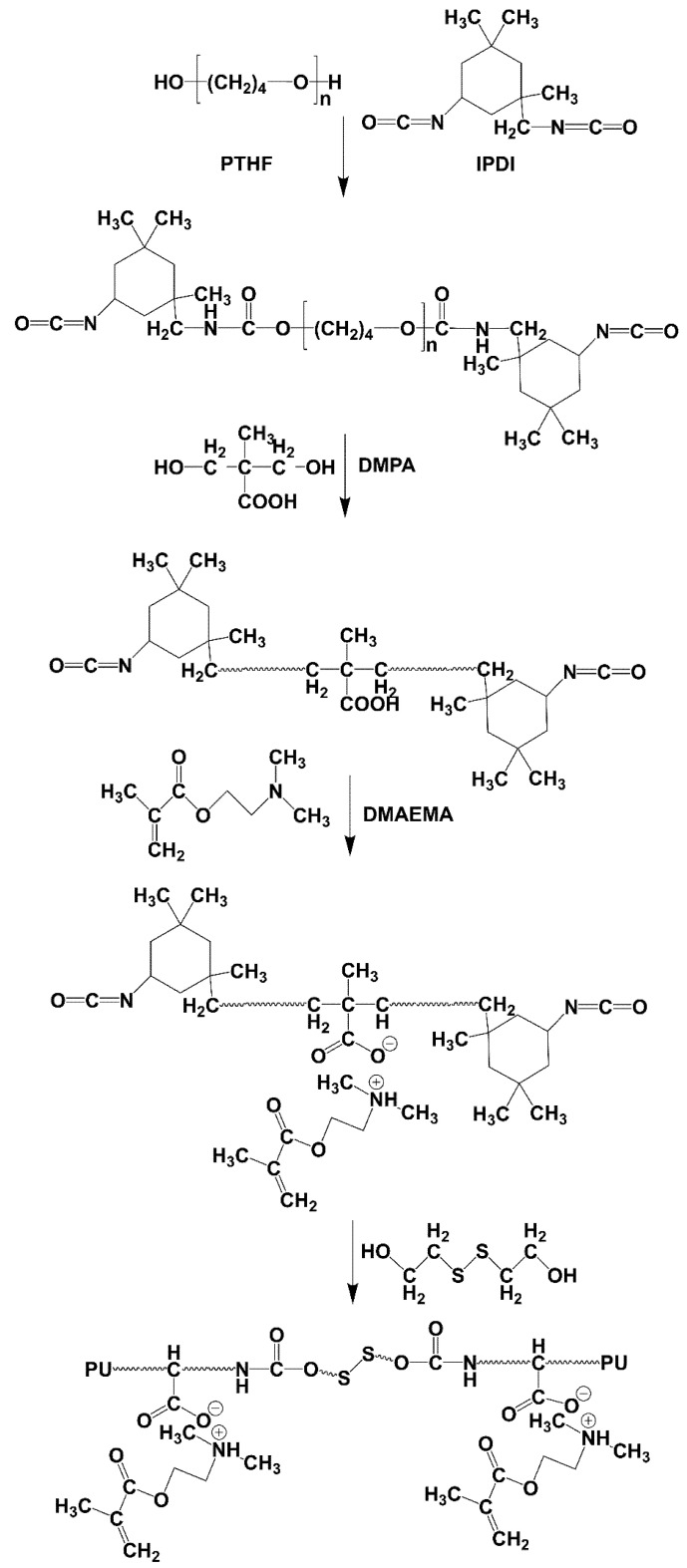
Preparation process of MFWPU prepolymer.

In the first reaction stage, dehydrated PTHF (25.60 g; Shanghai Aladdin Biochemical Technology Co., Ltd., Shanghai, China) was added to a four-neck flask in a dry nitrogen environment, and then 17.00 g of IPDI (Shanghai Heshibi Chemical Reagent Co., Ltd., Shanghai, China) and a small amount of DBTDL (Tianjin Fuchen Chemical Reagent Co., Ltd., Tianjin, China) were added to the system. The reaction was maintained at 80 °C for 1 h. In the second reaction stage, 3.97 g of DMPA (Shandong Wanduofu Chemical Reagent Co., Ltd., Zibo, China) and 10.00 g of DMF (Tianjin Fuchen Chemical Reagent Co., Ltd., Tianjin, China) were premixed at room temperature, and the mixed solution was added to the system. The reaction system was maintained at 80 °C for 1.5 h and then cooled to 60 °C. In total, 4.56 g DMAEMA (Shanghai Aladdin Biochemical Technology Co., Ltd., Shanghai, China) was added dropwise to the reaction system and maintained for 1 h, which was the key stage in the whole synthesis process. Then, 2,2′-disulfide diethanol (6.90 g, AlfaAesar Chemical Co., Ltd., Shanghai, China) was added to the reaction system and continued to react for 1 h. The MFWPU prepolymer synthesized in the four-neck flask was transferred to a plastic beaker. Subsequently, deionized water (80.01 g; Tianjin Jintongyuan Universal Water Center, Tianjin, China) was added dropwise to the MFWPU prepolymer at 1200 rpm for dispersion. Finally, the mixture continued to disperse for another 1.5 h to form MFWPU dispersion. The preparation process of the MFWPU prepolymer is displayed in Figure 1.

#### 2.2.2. Preparation of Waterborne Polyurethane (WPU-T) Using TEA as Neutralizer

The difference in the preparation procedure between WPU-T and MFWPU was that TEA (2.94 g; Shanghai Aladdin Biochemical Technology Co., Ltd., Shanghai, China) was used instead of DMAEMA (4.56 g; Shanghai Aladdin Biochemical Technology Co., Ltd., Shanghai, China) as the neutralizer of the system.

#### 2.2.3. Preparation of Waterborne Polyurethane (WPU) without Dynamic Disulfide Bond

During the preparation of WPU, 2,2′-disulfide diethanol was not used as a chain extender for the system, while BDO (4.03 g; Shanghai Aladdin Biochemical Technology Co., Ltd., Shanghai, China) was added as the chain extender in the final dispersion process. Other preparation processes were the same as MFWPU.

#### 2.2.4. Synthesis of P(CTFE-*alt*-IBVE) Latex

A typical experimental procedure is shown below. The 80.00 g of deionized water (Tianjin Jintongyuan Universal Water Center, Tianjin, China) was added to a 250 mL autoclave and then NaHCO_3_ (0.20 g; Tianjin Fuchen Chemical Reagent Co., Ltd., Tianjin, China), KPS (0.06 g; Traditional Chinese Medicine Group Chemical Reagent Co., Ltd., Shanghai, China), SDS (0.15 g; Shanghai Aladdin Biochemical Technology Co., Ltd., Shanghai, China) and IBVE (5.00 g; Shanghai Aladdin Biochemical Technology Co., Ltd., Shanghai, China) were added separately. A 250 mL autoclave equipped with a rupture disk (3000 psi), pressure gauge, inlet and outlet valves and a mechanical stirrer was pressurized with 20 bars of nitrogen to check the sealing feature of the reactor. The autoclave was then degassed until near vacuum (10^−2^ mbar) to remove oxygen. Finally, CTFE (Jiangsu Blue Planet Environmental Protection Technology Co., Ltd., Changzhou, China) monomer was added via double weighing (i.e., the weight difference before and after charging CTFE gas). The reaction was carried out with mechanical stirring at 60 °C for 8 h. After the reaction was completed, the autoclave was cooled and degassed to get rid of the unreacted monomers.

#### 2.2.5. Preparation of P(CTFE-*alt*-IBVE)/MFWPU Composite Particles

**Scheme 1:**
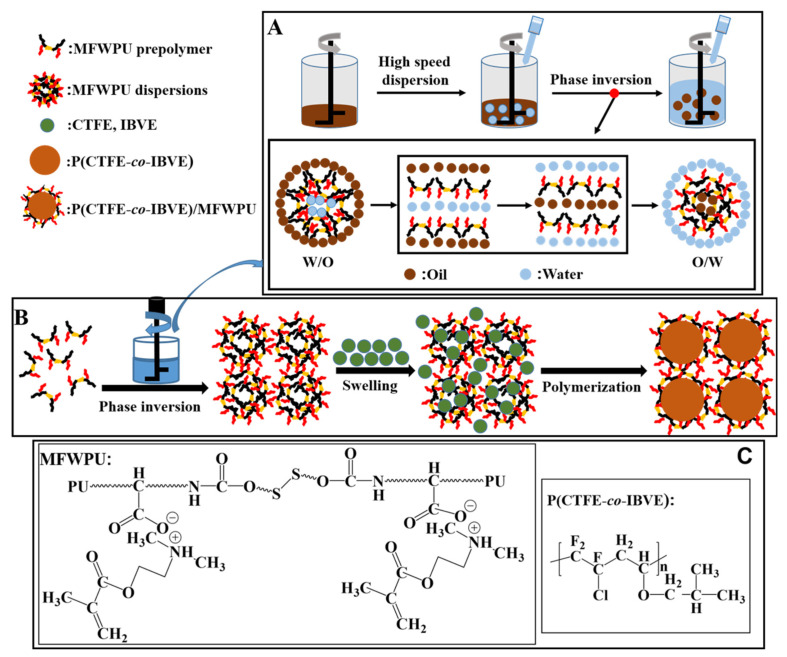
The synthesis diagram of P(CTFE-*alt*-IBVE)/MFWPU composite particles. (**A**) The phase inversion self-emulsification process of the MFWPU dispersion; (**B**) The synthesis diagram of P(CTFE-*alt*-IBVE)/MFWPU composite particles; (**C**) The structural formula of MFWPU.

Core–shell P(CTFE-*alt*-IBVE)/MFWPU composite particles were prepared with soap-free emulsion polymerization using MFWPU dispersion as a stabilizer and microreactor and CTFE and IBVE as comonomers, as shown in Scheme 1. Firstly, 1.00 g of latex containing MFWPU particles was diluted with 80.00 g deionized water (Tianjin Jintongyuan Universal Water Center, Tianjin, China) in a 250 mL flask, and the mixed solution was ultrasonicated for 30 min. The dispersion solution prepared above was added to the 250 mL autoclave equipped with bursting discs (3000 psi), pressure gauges, inlet and outlet valves and mechanical agitators, and then 0.20 g of NaHCO_3_ (Tianjin Fuchen Chemical Reagent Co., Ltd., Tianjin, China), 0.06 g of KPS (Traditional Chinese Medicine Group Chemical Reagent Co., Ltd., Shanghai, China) and 5.00 g of IBVE (Shanghai Aladdin Biochemical Technology Co., Ltd., Shanghai, China) were added to the system. The tightness of the autoclave was checked with 2 bar nitrogen. The autoclave was then placed under vacuum (10^−2^ mbar) to remove oxygen. Finally, CTFE monomer was added via double weighing (i.e., the weight difference before and after charging CTFE gas). The reaction was carried out under mechanical stirring at 60 °C for 8 h. After the reaction was completed, the autoclave was cooled and degassed to release the unreacted monomers.

#### 2.2.6. Preparation of P(CTFE-*alt*-IBVE)/MFWPU Composite Latex Coating

Substrates such as glass sheets and tinplate were thoroughly cleaned with alcohol (Shanghai Macklin Biochemical Technology Co., Ltd., Shanghai, China) and alkaline detergent, respectively and then rinsed off with deionized water. After the substrate was completely dried, the prepared latex was coated on the substrate with the drop-coating method and dried at 50 °C for about 24 h to obtain P(CTFE-*alt*-IBVE)/MFWPU composite latex coating.

### 2.3. Characterization

#### 2.3.1. Fourier Transform Infrared Spectroscopy (FTIR)

FTIR spectra were conducted on a Bruker Tensor-27 spectrometer (Bruker Company, Saarbrucken, Germany) to analyze the chemical structure of obtained products. The dehydrated samples were dissolved evenly in acetone, coated on a potassium bromide plate with a clean glass rod and then placed in a special infrared oven for drying.

#### 2.3.2. Dynamic Light Scattering (DLS) Measurement

The average particle diameter and size distribution of the MFWPU dispersion and core–shell P(CTFE-*alt*-IBVE)/MFWPU composite particles were tested with a dynamic light scattering particle size analyzer (Nano-ZS90 Zetasizer, Malvern, UK). A drop of latex was diluted with distilled water to prepare the samples for DLS. Subsequently, a transparent suspension was obtained and further ultrasonicated for 40 min.

#### 2.3.3. Scanning Electron Microscopy (SEM)

Morphology of P(CTFE-*alt*-IBVE)/MFWPU composite particles was characterized using a scanning electron microscopy (FE-SEM, Nano 450, operated at 10 kV, FEI Company, Hillsboro, OR, USA). Energy-dispersive X-ray spectroscopy-scanning electron microscopy (EDX-SEM) mapping was performed on the same SEM.

#### 2.3.4. X-ray Photoelectron Spectroscopy (XPS)

The chemical composition of the sample surface was investigated by XPS (ESCALAB 250Xi, Thermo Fisher Scientific, Waltham, MA, USA).

#### 2.3.5. UV Irradiation

The coating was placed under a 365 nm UV lamp with a power of 30 W (Jiangsu Haimen Qimbeier Instrument Manufacturing Co., Ltd., Haimen, China). The sample was located 0.14 m from the light source.

#### 2.3.6. Wetting Test

Static contact angles (CAs) of water droplets on the coating surfaces were measured using a DSA 30 S apparatus (Krüss Co., Hamburg, Germany). The static CAs were the mean values of 5 measurements with 4 μL water droplets at different places on each sample surface.

#### 2.3.7. Adhesion Test

For the adhesion test, the P(CTFE-*alt*-IBVE) latex and P(CTFE-*alt*-IBVE)/MFWPU composite latex were coated on a glass or tinplate substrate. The coating was cut into a grid pattern (10 × 10) with a uniform force by a cutting tool, and the blade just penetrated the coating to reach the substrate. A soft brush was used to gently brush the grid forward and backward 5 times. A piece of 3 M tape was adhered to 100 small squares and pressed evenly to ensure adhesion to the test piece, and then, the tape was quickly peeled off. Finally, the grid area on the substrate was enlarged using a magnifying glass to observe the coating of 100 squares clearly after peeling.

#### 2.3.8. Abrasion Analysis

A JM-IV wear Taber (Tianjin Yonglida Experimental Equipment Co., Ltd., Tianjin, China), designed and manufactured according to the national standard, GB/T1768-93, was arranged on a rotary disc using two grinding wheels. When the rotary wheel rotated, the left side of the grinding wheel was rubbed by the center of the extroverted template surface. The right side of the grinding wheel rose due to friction from the template surface center on the outside. During the turntable rotating cycle, the wear on the surface of the plate alternatively took place on the X-shaped intersecting rings. In this way, the surface of the template would be rubbed in all directions. The load on each grinding wheel was 250 g, and a Hertzian cylinder–plane contact pressure of about 12.1 MPa was estimated. This test method was chosen largely due to its well-assessed and standardized approach.

#### 2.3.9. Scratch Self-Healing Test

Scratch self-healing test was evaluated based on the scratch recovery degree at different temperatures and times. The surface of the coating was scratched with a blade, and then the self-healing of the coating’s scratch was observed and recorded at room temperature (25 °C) and 80 °C.

#### 2.3.10. Thermogravimetric Analysis (TGA)

TGA is an important method of evaluating the thermal stability of polymers. In this work, TGA was performed using an SDT/Q-600 analyzer (TA Instruments, New Castle County, DE, USA) with a nitrogen flow of 100 mL/min to investigate the thermal stability of composite latex coatings.

#### 2.3.11. Dynamic Thermomechanical Analysis (DMA)

The glass transition temperature (*T*_g_) of composite latex particles was measured on DMA (Tritec 2000, Triton technology, Chelmsford, UK).

#### 2.3.12. Gel Permeation Chromatography (GPC)

The number average molecular weight (*M*_n_) and the polydispersity index (PDI) of the MFWPU were measured with GPC on a Waters 1515 apparatus (Waters Company, Milford, MA, USA).

#### 2.3.13. Nuclear Magnetic Resonance (NMR)

The ^1^H NMR spectra were characterized on a Bruker Avance III HD Nuclear Magnetic Resonance (NMR) spectrometer.

#### 2.3.14. Theoretical Fluorine Content

Theoretical fluorine content is calculated by the following formula:

(1) $$F\mathrm{wt}.\%=\frac{Mass\;of\;CTFE\times 0.489\;\left(F\%\;of\;CTFE\right)}{Total\;mass\;of\;CTFE+IBVE+MFWPU}$$

where *F wt.*% represents the theoretical fluorine content. Mass of CTFE × 0.489 (*F*% of CTFE) is the total mass of the F element added to the system. Total mass of CTFE + IBVE + MFWPU refers to the total mass of the monomer and stabilizer added to the system.

## 3. Results and Discussion

### 3.1. Particle Size and Composition of the MFWPU and P(CTFE-alt-IBVE)/MFWPU Composite Particles

Multifunctional waterborne polyurethane (MFWPU) prepolymers containing dynamic S-S and C=C were prepared from PTHF and IPDI using DMPA and 2,2′-disulfide diethanol as chain extenders and DMAEMA as a neutralizer. The preparation process of the MFWPU prepolymer is displayed in Figure 1. Water droplets were uniformly added to the amphiphilic MFWPU prepolymer under high-speed dispersion. The MFWPU dispersion was obtained through the phase inversion self-emulsification process of the system from water-in-oil (W/O) to oil-in-water (O/W) (Scheme 1A). The structural formula of the MFWPU is shown in Scheme 1C. MFWPU dispersion can exist stably in a water system owing to its amphipathy and can provide reaction sites for comonomers. P(CTFE-*alt*-IBVE)/MFWPU composite particles were prepared with soap-free emulsion polymerization using MFWPU dispersion as a stabilizer and microreactor and CTFE and IBVE as comonomers, as shown in Scheme 1B.

**Figure 2:**
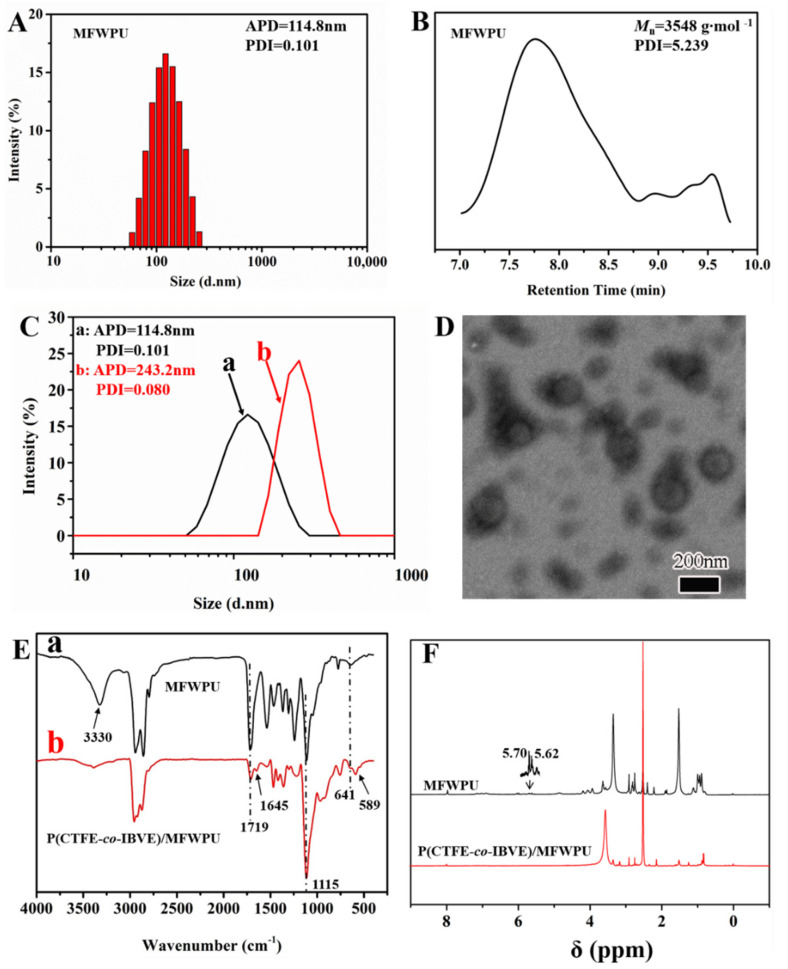
(**A**) Particle size distribution histogram and (**B**) GPC curve of MFWPU. (**C**) Particle size distribution of MFWPU (**a**) and P(CTFE-*co*-IBVE)/MFWPU composite particles (**b**). (**D**) SEM micrograph of P(CTFE-*co*-IBVE)/MFWPU composite particles. (**E**) FTIR spectra of MFWPU (**a**) and P(CTFE-*co*-IBVE)/MFWPU composite particles (**b**). (**F**) ^1^H NMR spectra of MFWPU and P(CTFE-*co*-IBVE)/MFWPU composite particles.

Figure 2A displays the particle size distribution histograms of MFWPU. It can be seen from Figure 2A that the average particle diameter (APD) of MFWPU is about 114.8 nm, with the polydispersity index (PDI) being 0.101. The molecular weight of MFWPU was determined with GPC. As shown in Figure 2B, the number average molecular weight (*M*_n_) of MFWPU oligomer is 3548 g·mol^−1^, and the PDI is 5.239. Generally, a polymer with a number average molecular weight between 10,000 and 1000 is called an oligomer. Consequently, the prepared MFWPU is an oligomer. Moreover, the chemical composition and structure of MFWPU are characterized by FTIR in Figure 2E.

It can be seen from Figure 2C that the particle sizes of the MFWPU and P(CTFE-*alt*-IBVE)/MFWPU composite particles are 114.8 nm and 245.1 nm, with the PDI being 0.101 and 0.080, respectively. P(CTFE-*alt*-IBVE)/MFWPU composite particles present a larger particle size and a narrower distribution compared with those of MFWPU. This is because during the preparation of P(CTFE-*alt*-IBVE)/MFWPU composite particles hydrophobic CTFE and IBVE monomers swell in the MFWPU microreactor under mechanical stirring. Then, the initiator decomposes to form free radicals under the heating of the system and diffuses into the MFWPU microreactor. Finally, the monomer is initiated for polymerization. Therefore, the particle size of P(CTFE-*alt*-IBVE)/MFWPU composite particles is significantly larger than that of MFWPU. Figure 2D is the SEM micrograph of the P(CTFE-*alt*-IBVE)/MFWPU composite particles. It can be seen that the composite particles are spherical. Some particles adhered together due to the low glass transition temperature of the shell polyurethane and the easy movement of the molecular chain. In order to further identify the chemical composition of MFWPU and P(CTFE-*alt*-IBVE)/MFWPU composite particles, FTIR spectra were measured, as seen in Figure 2E. As is shown in the spectrum of the MFWPU in Figure 2(Ea), the peak at 1115 cm^−1^ is the stretching vibration of C-O-C. The characteristic peak at 1719 cm^−1^ can be attributed to the deformation vibration of O-C=O. The peak at 3330 cm^−1^ can be ascribed to the stretching vibration of -NH-COO-. The peak at 641 cm^−1^ can be attributed to the characteristic peak of S-S. All these peaks indicate that MFWPU was synthesized successfully. Peaks at 589 cm^−1^ and 1645 cm^−1^ in Figure 2(Eb) can be assigned to the CF_2_ stretching vibration in the P(CTFE-*alt*-IBVE). In conclusion, all the characterizations above demonstrate that the P(CTFE-*alt*-IBVE)/MFWPU composite particles were prepared successfully.

### 3.2. Influence of 2,2′-Disulfide Diethanol Incorporation on Scratch Self-Healing Performance of the Composite Coating

**Figure 3:**
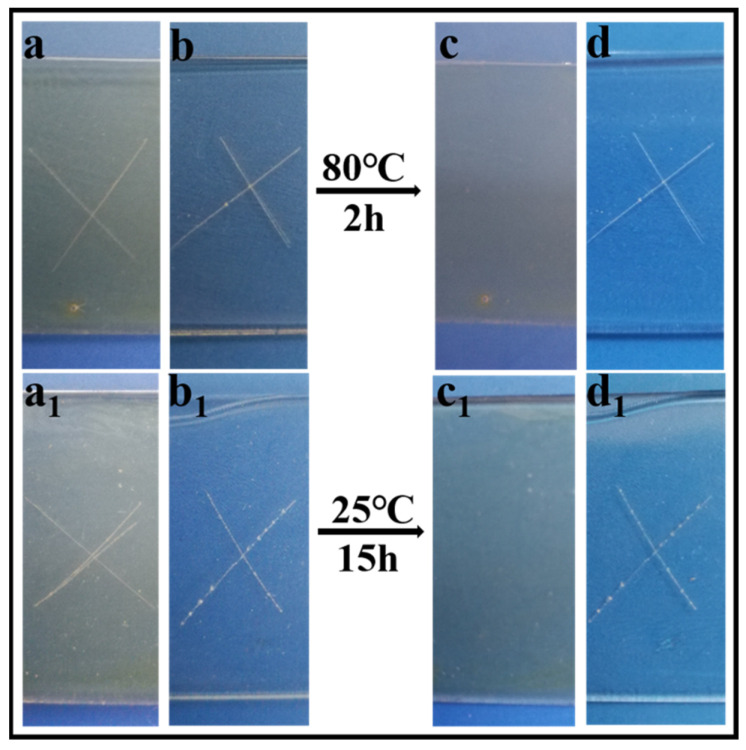
Self-healing performance: Coating scratch images of P(CTFE-*alt*-IBVE)/MFWPU (**a**,**a_1_**), P(CTFE-*alt*-IBVE)/WPU (**b**,**b_1_**), P(CTFE-*alt*-IBVE)/MFWPU (**c**) and P(CTFE-*alt*-IBVE)/WPU (**d**) at 80 °C for 2 h and P(CTFE-*alt*-IBVE)/MFWPU (**c_1_**) and P(CTFE-*alt*-IBVE)/WPU (**d_1_**) at 25 °C for 15 h.

The MFWPU dispersion containing 2,2′-disulfide diethanol and the WPU dispersion without 2,2′-disulfide diethanol were prepared to produce P(CTFE-*alt*-IBVE)/MFWPU and P(CTFE-*alt*-IBVE)/WPU for the final preparation of the composite coatings. Figure 3 displays the scratch situation images of the P(CTFE-*alt*-IBVE)/MFWPU and P(CTFE*alt*-IBVE)/WPU coatings before and after placement at 80 °C and 25 °C. It can be seen from Figure 3(c,c_1_) that the surface scratches on the P(CTFE-*alt*-IBVE)/MFWPU coatings healed automatically after the coatings were placed at 80 °C for 2 h and 25 °C for 15 h, while the surface scratches on the P(CTFE-*alt*-IBVE)/WPU coatings did not self-heal in same conditions, as shown in Figure 3(d,d_1_), which illustrates that the existence of dynamic S-S in 2,2′-disulfide diethanol plays a key role in the self-healing process of coating scratches. The self-healing mechanism of P(CTFE-*alt*-IBVE)/MFWPU coatings will be introduced in detail in Figure 5, below. On the one hand, the sulfhydryl group can be obtained after the disulfide bond breaks, and the disulfide bond can be formed again by connecting the sulfur atoms in the same or different disulfide bonds through an oxidation reaction in the sulfhydryl group [35]. On the other hand, it can also be seen that the self-healing time of the coating scratch is significantly shortened with the increase in the treatment temperature, implying that the movement rate of the molecular chain segment obviously affects the self-healing speed.

### 3.3. Influence of C=C Bond in MFWPU on Surface Element Composition of the Composite Coatings

**Figure 4:**
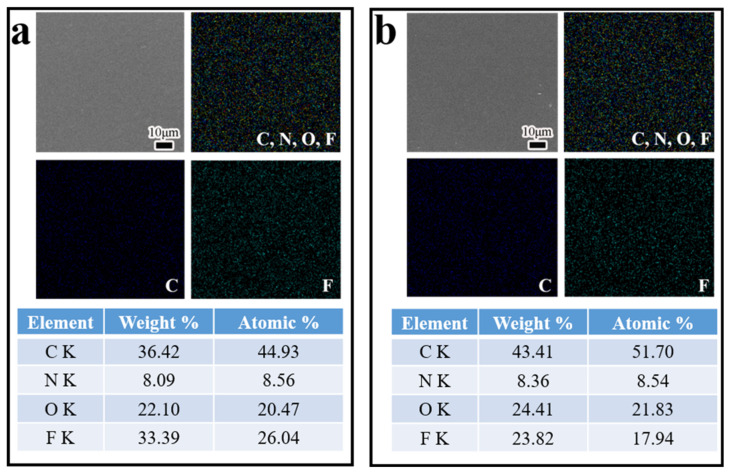
EDX-SEM micrographs of P(CTFE-*alt*-IBVE)/WPU-T (**a**) and P(CTFE-*alt*-IBVE)/MFWPU (**b**) coatings prepared, respectively, using TEA and DMAEMA as neutralizers.

To evaluate the influence of the predesigned C=C bond in the MFWPU on the surface chemicals of the composite coatings, the P(CTFE-*alt*-IBVE)/WPU-T and P(CTFE-*alt*-IBVE)/MFWPU coatings obtained, respectively, using TEA and DMAEMA neutralizers were further investigated. The distributions of the C, N, O and F elements on both coating surfaces are displayed in EDX-SEM micrographs (Figure 4). It can be concluded that the relative weight percentage the of F element on the surface of the composite coating prepared with TEA as a neutralizer is 33.39%, which is larger (23.82%) than with DMAEMA as a neutralizer. This can be explained by the C=C bond in the DMAEMA molecular structure. During the preparation of P(CTFE-*alt*-IBVE)/MFWPU composite particles via the reactive MFWPU, with DMAEMA as a neutralizer, the C=C bond in the MFWPU will react with CTFE and IBVE monomers. As shown in Figure 2F, the weak signal at 5.0–6.0 ppm is assigned to the -C=CH_2_ in the ^1^H NMR spectrum of the MFWPU, while there is no -C=CH_2_ signal in P(CTFE-*alt*-IBVE)/MFWPU, which further confirms the fact that the -C=CH_2_ functional group in the MFWPU has reacted with CTFE and IBVE monomers. Thus, the compatibility between the MFWPU shell and the P(CTFE-*alt*-IBVE) core increases. It is widely known that the fluorine-containing segments located inside the coating have low surface energy and tend to migrate to the coating surface during the film-forming process of composite latex [16], thereby resulting in the occurrence of phase separation. However, it is worth mentioning that the chemical bonding interaction of MFWPU and the core polymer reduces the phase separation between the shell and the core and decreases the migration tendency of fluorine-containing segments located inside the coating near the coating surface. Thus, the relative weight percentage of the F element on the surface of the composite coating, achieved with DMAEMA as a neutralizer, is less than that of TEA as a neutralizer. Simultaneously, fluorine-containing domains (P(CTFE-*alt*-IBVE)) can be evenly distributed inside the P(CTFE-*alt*-IBVE)/MFWPU coating.

**Figure 5:**
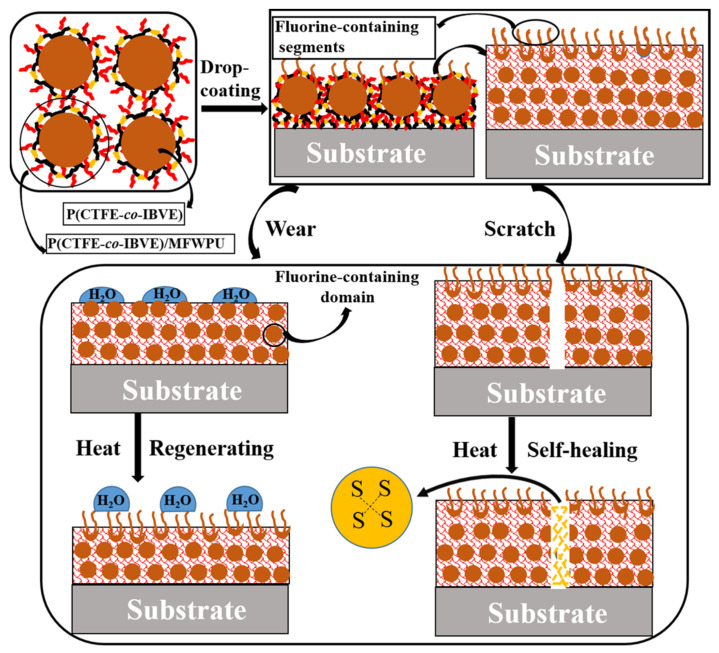
A double self-healing model of P(CTFE-*alt*-IBVE)/MFWPU composite coating.

Furthermore, the phenomenon where the relative weight percentage of the F element on the surface of the composite coating prepared with DMAEMA as a neutralizer is larger than the theoretical fluorine content (16.38%) (calculated in Formula (1)) of the coating also reveals that, during the film-forming process, the fluorine-containing segments in the core layer of the composite particles located on the coating surface have low surface energy compared with the shell polymer, and they will be more inclined to arrange at the interface between the coating and air, thereby enriching the coating surface. Finally, a composite coating with enriched fluorine-containing segments on the surface and a uniform distribution of fluorine-containing domains inside can be obtained, as shown in Figure 5.

### 3.4. Double Self-Healing Model of Composite Coating

Based on the above research on the effects of dynamic S-S and C=C in MFWPU on the scratch self-healing performance and surface element composition of the composite coatings, a coating scratch–friction double self-healing model is proposed (Figure 5). On the one hand, during the film-forming process, the fluorine-containing segments with low surface energy in the core layer of the composite particles located on the coating surface tend to arrange at the interface between the coating and air, thereby enriching the coating surface. For the composite particles located inside the coating, the chemical bonding interaction between the MFWPU and comonomers increases the compatibility between the shell polymer and core polymer and further confines the migration and aggregation of the fluorine-containing segments in the fluorine-containing domains located inside the coating near the coating surface during the film-forming process of composite latex. Ultimately, the composite coating is obtained with the enriched fluorine-containing segments on the surface and the uniform distribution of fluorine-containing domains inside. When the surface of the coating is worn, fluorine-containing segments enriched on the coating surface are also reduced, and then, the new surface layer will be exposed. The fluorine-containing segments in the fluorine-containing domains located on the newly formed coating surface could rearrange due to the acceleration of the molecular chain movement as the coatings are heated so as to supplement the fluorine-containing segments for the newly formed coating surface, achieving the purpose of element regeneration on the coating surface. Thus, the initial hydrophobicity and weather resistance of the coating are restored. This self-healing process can be repeated because the fluorine-containing domains are evenly distributed inside the coating. On the other hand, when the coating suffers from physical scratches, the scratches can heal and recover to the original state based on the exchange reaction of the dynamic S-S in 2,2-disulfide diethanol and the movement of molecular segments under heating conditions, which is called the self-healing characteristic. In summary, the double self-healing model of P(CTFE-*alt*-IBVE)/MFWPU composite coatings is proposed: the coating can achieve a self-healing effect when suffering from surface wear and physical scratches so as to prolong the life of coatings and reduce the repair cost.

### 3.5. Regeneration Performance of the Composite Coating Surface

**Figure 6:**
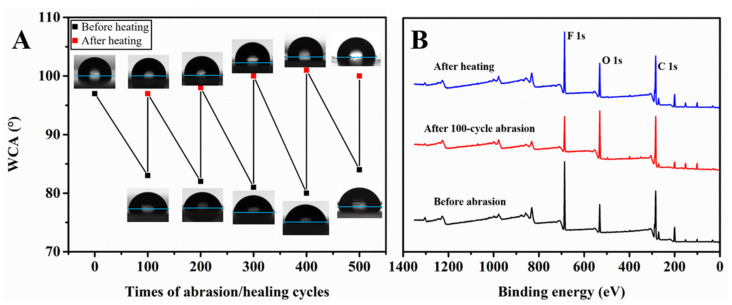
(**A**) Changes in WCAs after repeated abrasion and heat treatment at 80 °C for 2 h; (**B**) XPS spectra of P(CTFE-*alt*-IBVE)/MFWPU composite coating surface before abrasion after 100 abrasion cycles and after heating.

To further verify the double self-healing model of the P(CTFE-*alt*-IBVE)/MFWPU composite coating in Figure 5, the surface regeneration performance of the coating prepared with the mass feed ratio of the MFWPU and IBVE—being 1:5 after abrasion and heat treatment—was investigated. All water contact angles (WCA) tests of the coating were conducted after every 100 abrasion and heating cycles (Figure 6A). The WCA of the coating decreased after every 100 abrasion cycles. After heating at 80 °C for 2 h, the original hydrophobicity of the coating could be recovered. Furthermore, the coating presents a good regeneration ability even after 5 cycles of 100 abrasion and subsequent heat treatments.

To study the self-healing behavior of the coating surface after abrasion and heating at 80 °C for 2 h, the surface elements of the coating were characterized with XPS. It can be seen from Figure 6B that the peak intensity of the F1s decreases significantly after 100 abrasion cycles compared with the intensity before abrasion. Subsequently, the coating was heated at 80 °C for 2 h, and the peak intensity of the F1s increased to a value close to the original state. Moreover, the hydrophobicity of the coating returned, generally, to the state before abrasion. It can be inferred that the fluorine content on the surface of the coating directly affects the performance of the coating. The fluorine-containing segments on the coating surface will be worn off when the coating is subjected to physical abrasion. Afterward, the decrease in fluorine content on the coating surface leads to an increase in the coating surface energy so that the coating tends to be easily wetted by water droplets and changes from hydrophobicity to hydrophilicity. In view of the uniform distribution of fluorine-containing domains inside the coating, the fluorine-containing segments in the fluorine-containing domains located on the newly formed coating surface can rearrange due to the acceleration of molecular chain movement after heating at 80 °C for 2 h, resulting in an increase in the fluorine content of the newly formed coating surface. This self-healing process can be repeated. Hence, the fluorine element on the surface of the coating achieves regeneration so that the coating returns to the original hydrophobicity, which further verifies the usefulness of the proposed double self-healing model of the P(CTFE-*alt*-IBVE)/MFWPU composite coating (Figure 5).

### 3.6. Optimization of Two Contradictory Properties of Scratch Self-Healing Polymers with Respect to Efficient Recovery and Durability

Efficient recovery and durability (including thermal stability, water resistance and so on) are regarded as the most important properties of scratch self-healing polymers [21]. In this work, the scratch self-healing performance of the coating mainly depends on the introduction of dynamic S-S and the movement of molecular chain segments. Therefore, the increase in MFWPU component content in the system can provide the composite coating with excellent scratch self-healing performance, but the thermal stability and water resistance of the composite coating will also be weakened due to the characteristics of WPU. This implies that these two characteristics are often contradictory, so it is difficult to optimize them simultaneously. In order to optimize both the scratch self-healing properties and the durability, the influences of P(CTFE-*alt*-IBVE)/MFWPU composite particles with different core–shell mass feed ratios on the scratch self-healing properties, thermal stability and water resistance of the coatings were further investigated.

#### 3.6.1. P(CTFE-*alt*-IBVE)/MFWPU Composite Particles Prepared with Different Mass Feed Ratios of MFWPU and IBVE

**Figure 7:**
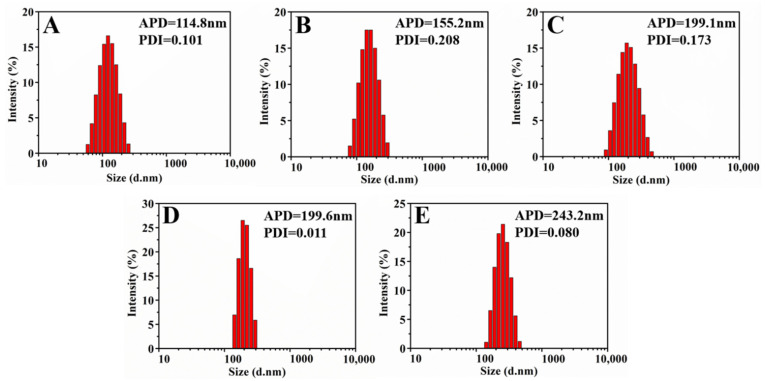
The average particle size distributions of MFWPU (**A**) and P(CTFE-*alt*-IBVE)/MFWPU composite particles prepared with MFWPU versus IBVE mass feed ratios of 1:1 (**B**), 1:3 (**C**), 1:4 (**D**) and 1:5 (**E**).

IBVE cannot undergo free radical homopolymerization, but it can react with CTFE to form an alternating copolymer (P(CTFE-*alt*-IBVE)) [21]. A series of P(CTFE-*alt*-IBVE)/MFWPU composite particles with different core–shell mass feed ratios were prepared by maintaining the amount of CTFE excess but changing the additional amount of IBVE monomer. It can be seen from Figure 7 that the average particle size of the MFWPU is 114.8 nm. When the mass feed ratios of MFWPU and IBVE are 1:1, 1:3, 1:4 and 1:5, the corresponding average particle sizes of P(CTFE-*alt*-IBVE)/MFWPU are 155.2 nm, 199.1 nm, 199.6 nm and 243.2 nm, respectively. The reason is that the number of MFWPU microreactors formed in the aqueous solution remains unchanged when the additional amount of MFWPU is constant. With the additional amount of monomer increases, the amount of both swelling comonomers and polymerization comonomers in every MFWPU microreactor increases, resulting in the average particle size of the composite particles increasing gradually. However, when the added IBVE monomer continues to increase, the content of P(CTFE-*alt*-IBVE) in the system also continues to increase, which enhances the hydrophobicity of the composite particles. As a result, the imbalance of the hydrophilicity and hydrophobicity of the system leads to demulsification.

#### 3.6.2. Influence of Different MFWPU/IBVE Mass Feed Ratios on the Scratch Self-Healing Performance of the Composite Coatings

**Figure 8:**
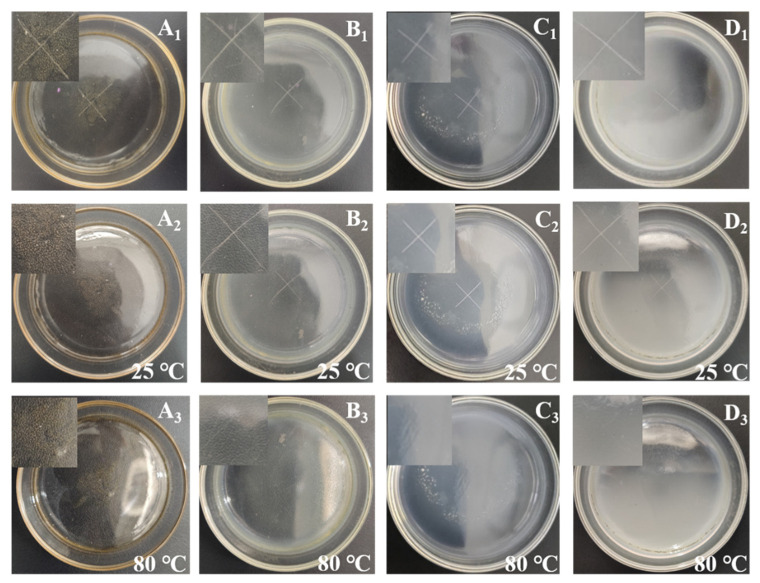
Images of a self-healing performance test for a coating scratch: P(CTFE-*alt*-IBVE)/MFWPU composite coatings prepared with different mass feed ratios of MFWPU versus IBVE: 1:1 (**A**), 1:3 (**B**), 1:4 (**C**) and 1:5 (**D**) at the initial time (**A_1_**–**D_1_**), 15 h at 25 °C (**A_2_**–**D_2_**) and 2 h at 80 °C (**A_3_**–**D_3_**).

The P(CTFE-*alt*-IBVE)/MFWPU composite particles synthesized through different mass feed ratios of MFWPU versus IBVE were further used to prepare the corresponding coatings. The coatings were scratched with a blade, as seen in Figure 8(A_1_–D_1_), corresponding to 1:1, 1:3, 1:4 and 1:5 ratios of MFWPU versus IBVE, and the scratch self-healing performance was investigated. It can clearly be observed from Figure 8(A_2_) that the scratches on the composite coating prepared with a 1:1 mass feed ratio of MFWPU versus IBVE obviously healed automatically after 15 h at 25 °C, while the scratches on the other mass feed ratio coatings did not present significant changes under this condition (Figure 8(B_2_–D_2_)). Whereas the heat treatment was performed at 80 °C for 2 h, the composite coatings exhibited obvious self-healing performance, and the scratches on all the coatings disappeared, as shown in Figure 8(A_3_–D_3_). On the one hand, with the increase in the P(CTFE-*alt*-IBVE) component content in the composite particles, the content of the MFWPU component decreased relatively; therefore, the content of the dynamic S-S in the system also decreased relatively. On the other hand, with respect to the P(CTFE-*alt*-IBVE)/MFWPU core–shell particles, the *T*_g_ of the core is higher than that of the shell. With the content of the core polymer increasing, the movement of the molecular chain becomes difficult. Therefore, a higher temperature is required to promote the movement of the molecular chain to achieve the purpose of scratch self-healing.

**Figure 9:**
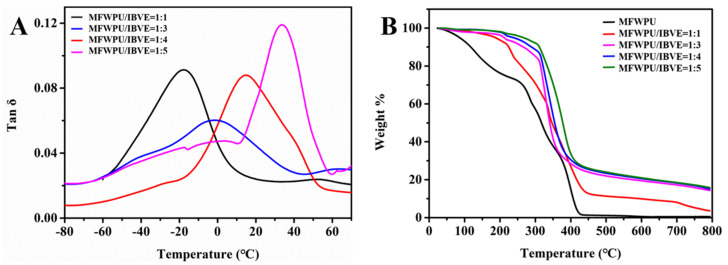
(**A**) TGA curves of MFWPU and P(CTFE-*alt*-IBVE)/MFWPU prepared with different mass feed ratios of MFWPU and IBVE; (**B**) DMA spectra of P(CTFE-*alt*-IBVE)/MFWPU composite coatings prepared with different mass feed ratios of MFWPU versus IBVE.

To further study the effect of different MFWPU/IBVE mass feed ratios on the *T*_g_ of P(CTFE-*alt*-IBVE)/MFWPU composite coatings and better understand the self-healing performance, DMA tests were carried out on composite coatings prepared with different IBVE additions, among which, the peak temperature of the tan δ curves were considered to be *T*_g_. As shown in Figure 9A, when the mass feed ratios of MFWPU versus IBVE were 1:1, 1:3, 1:4 and 1:5, the *T*_g_s of the corresponding composite coatings are −18 °C, −2 °C, 15 °C and 34 °C, respectively, which further demonstrates that the *T*_g_ of the P(CTFE-*alt*-IBVE)/MFWPU composite coating increases and gradually approaches to that of P(CTFE-*alt*-IBVE) (Figure S1) with the increase in P(CTFE-*alt*-IBVE) content. This is because the movement of the molecular chain in the P(CTFE-*alt*-IBVE) phase is more difficult compared with that in the MFWPU phase. As a result, with the increase in P(CTFE-*alt*-IBVE) content in the system, a higher temperature is expected to promote the movement of the molecular chain for the purpose of self-healing. Furthermore, only one peak can be observed for each tan δ curve in the four kinds of composite coatings, which further illustrates the occurrence of free radical copolymerization between C=C in MFWPU and the monomers of CTFE and IBVE, such that the MFWPU phase, together with the P(CTFE-*alt*-IBVE) phase, exhibits good compatibility.

#### 3.6.3. Influence of Different MFWPU/IBVE Mass Feed Ratios on the Thermal Stability of the Composite Coatings

Thermogravimetric analysis is usually used to investigate the thermal stability of materials. It can be seen from the TGA curves (Figure 9B) that the initial decomposition temperature of P(CTFE-*alt*-IBVE)/MFWPU composite coatings is significantly higher than that of the MFWPU. Moreover, the initial decomposition temperature of the P(CTFE-*alt*-IBVE)/MFWPU composite coatings increases when the additional amount of IBVE increases, the highest temperature being 307 °C when the mass feed ratio of MFWPU versus IBVE is 1:5. These phenomena indicate that the P(CTFE-*alt*-IBVE) phase can improve the thermal stability of the composite coating effectively, owing to the fact that the bond energy of F-C is the largest among all the chemical bonds, that is, 485 kJ/mol [12,13,14] Further, it can be seen from the TGA curve of the MFWPU that it almost decomposed completely at 426 °C, and the remaining carbon content was only 1.98%. When the mass feed ratio of MFWPU versus IBVE is 1:1, the weight loss trend of the composite coating is similar to that of the MFWPU. Nevertheless, its remaining carbon content is 16.94% at 426 °C, which is larger than that of the MFWPU due to the existence of P(CTFE-*alt*-IBVE). As the mass feed ratio of MFWPU versus IBVE continues to increase to 1:3, 1:4 and 1:5, the corresponding remaining carbon contents of the composite coatings at 426 °C are 25.62%, 27.26% and 27.91%, respectively, which are significantly higher than that of the mass feed ratio of MFWPU versus IBVE at 1:1. Furthermore, the weight loss trend of these three curves is significantly different compared with the MFWPU. The reason can be attributed to the decrease in relative MFWPU content, with the content of the P(CTFE-*alt*-IBVE) component increasing in the system. This can also illustrate the great compatibility of MFWPU and P(CTFE-*alt*-IBVE).

#### 3.6.4. Influence of Different MFWPU/IBVE Mass Feed Ratios on the Water Resistance of the Composite Coatings

**Figure 10:**
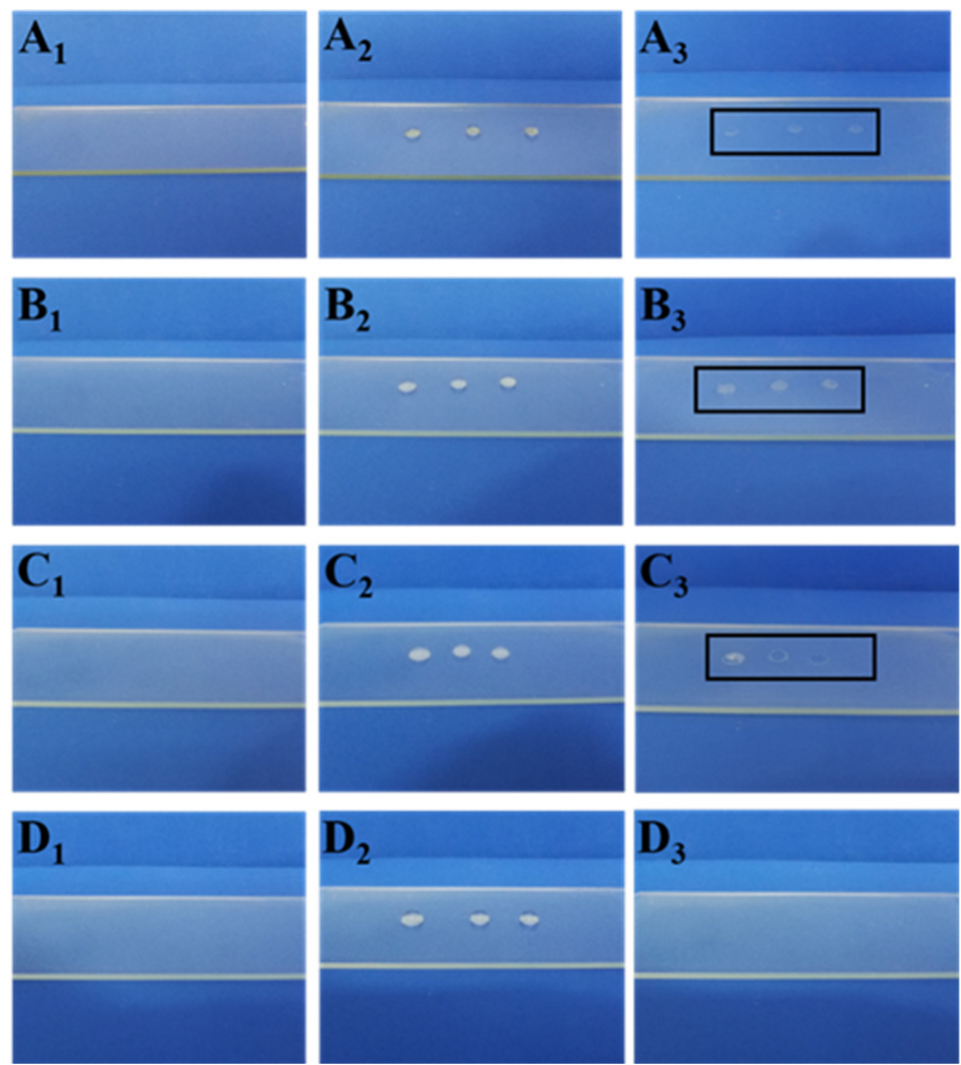
Coating water resistance test: images of P(CTFE-*alt*-IBVE)/MFWPU composite coatings prepared with different mass ratios of MFWPU and IBVE: (**A**) 1:1, (**B**) 1:3, (**C**) 1:4 and (**D**) 1:5 (**A_1_**–**D_1_**) before and (**A_2_**–**D_2_**) after dripping water drops and (**A_3_**–**D_3_**) water droplets infiltration for 24 h.

The water resistance of the coatings prepared with different mass feed ratios of MFWPU versus IBVE was further investigated. From A_1_, B_1_ and C_1_ to A_3_, B_3_ and C_3_ in Figure 10, it can be clearly seen that the coatings prepared with the mass feed ratio of MFWPU versus IBVE being 1:1, 1:3 and 1:4 swelled and dissolved in different degrees and turned white after water droplet infiltration for 24 h. However, the surface of the coating prepared with an MFWPU versus IBVE mass feed ratio of 1:5 presents excellent water resistance that has almost no changes after water droplet infiltration for 24 h. This can be ascribed to the increase in P(CTFE-*alt*-IBVE) content, with the amount of IBVE monomer increasing. Consequently, the existence of fluoropolymers endows the coatings with lower surface energy and effectively prevents the wetting and infiltration of water droplets so that the coatings present excellent water resistance.

In summary, when the mass feed ratio of MFWPU versus IBVE is 1:5, the prepared P(CTFE-*alt*-IBVE)/MFWPU composite coatings exhibit not only excellent scratch self-healing performance at 80 °C for 2 h but also good water resistance and thermal stability. The excellent scratch self-healing performance and durability of the coating will better extend their lifetimes and reduce maintenance costs.

### 3.7. UV Resistance and Adhesion Performance of Composite Coatings

**Figure 11:**
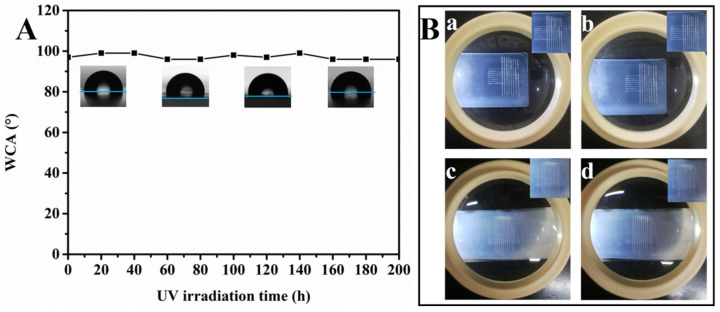
(**A**) WCAs of P(CTFE-*alt*-IBVE)/MFWPU composite coating under UV irradiation for different amounts of time. (**B**) Adhesion performance determination of the coatings via a cross-cut test: glass-based coating (**a**) and tinplate coating (**c**) before 3M tape application; glass-based coating (**b**) and tinplate coating (**d**) after 3M tape application.

Owing to the outdoor UV irradiation that coatings are usually exposed to, UV resistance has become an important performance criterion for the coatings. As shown in Figure 11A, the UV resistance of P(CTFE-*alt*-IBVE)/MFWPU composite coating prepared with an MFWPU and IBVE mass feed ratio of 1:5 was characterized by testing the WCAs of the coating under UV irradiation for different amounts of time. As expected, the P(CTFE-*alt*-IBVE)/MFWPU composite coating presented little change in WCA and good hydrophobicity even after it was exposed to UV irradiation for 200 h. This can be attributed to a large number of F-C bonds in P(CTFE-*alt*-IBVE). Therefore, the composite coating exhibits excellent UV resistance.

The P(CTFE-*alt*-IBVE) coating showed poor adhesion on glass substrates with grade 0B (Figure S2), while the prepared P(CTFE-*alt*-IBVE)/MFWPU composite coating achieved grade 5B on both glass and tinplate substrates, with good adhesion properties (Figure 10B). This can be explained by the fact that the low surface energy of the P(CTFE-*alt*-IBVE) coating leads to its poor adhesion to the substrate, while the addition of MFWPU with good adhesion properties endows the P(CTFE-*alt*-IBVE)/MFWPU composite coating with excellent adhesion. There are still many highly polar groups in polyurethane, which can bond with various substrates. For example, the urea bond formed by the reaction of the -NCO group in the polyurethane molecule and the water molecule can form a hydrogen bond with the surface oxide of the metal so that the polyurethane and substrate can be firmly bonded [37]. Consequently, the prepared P(CTFE-*alt*-IBVE)/MFWPU composite coating can be widely used on a variety of different substrates and shows good adhesion.

## 4. Conclusions

In summary, P(CTFE-*alt*-IBVE)/MFWPU composite latex particles were successfully prepared via soap-free emulsion copolymerization, and the corresponding coatings were further manufactured, in which MFWPU acted as the microreactor and stabilizer in the copolymerization process of CTFE and IBVE. This can not only replace traditional small molecule surfactants but also endows the coating with double self-healing performance and excellent adhesion at the same time. The introduction of a disulfide bond to MFWPU provides the composite coating with scratch self-healing performance, while the embedded C=C in the MFWPU increases the compatibility of the core–shell polymers and endows the surface elements of the composite coating with a self-healing or regeneration ability after abrasion and heat treatment cycles, which further verifies the usefulness of the double self-healing model of the coating. In addition, efficient recovery and durability, which are two contradictory characteristics of self-healing polymers, can be optimized so that a composite coating prepared with an MFWPU and IBVE mass feed ratio of 1:5 possesses excellent thermal stability, water resistance and double self-healing properties. The P(CTFE-*alt*-IBVE)/MFWPU composite latex particles show excellent synergistic effects between the components, and the coatings have good adhesion to a variety of substrates. Furthermore, the WCAs of the coatings hardly change even after high-intensity UV irradiation for 200 h. The composite coating can adapt to the harsh outdoor environment and is expected to be widely used in various protective coatings.

## Data Availability

The data presented in this work are available upon request from the corresponding author.

## Supplementary Materials

The following supporting information can be downloaded at: https://www.mdpi.com/article/10.3390/nano12234216/s1, Figure S1: DMA spectra of P(CTFE-alt-IBVE); Figure S2: Adhesion performance determination of the P(CTFE-*co*-IBVE) coating by cross-cut test: (A) glass-based coating before 3M tape application; (B) glass-based coating after 3M tape application.
