# Supplementary material for: Multifunctional Waterborne Polyurethane Microreactor-Based Approach to Fluorocarbon Composite Latex Coatings with Double Self-Healing and Excellent Synergistic Performances
Source: Nanomaterials (Basel). 2022 Nov 27;12(23):4216. doi: 10.3390/nano12234216 (PMC9737679; doi:10.3390/nano12234216)
Supplement: Supplementary file 1 [file nanomaterials-12-04216-s001.zip › nanomaterials-2049943-supplementary.pdf]

Supporting Information

Multifunctional Waterborne Polyurethane Microreactor-Based Approach to Fluorocarbon Composite Latex Coatings with Double Self-Healing and Excellent Synergistic Performances

Chao Li [1], Huimin Guo [1], Ning Zhang [1], Yao Jin [1], Kai Han [1], Jinfeng Yuan [1,2], Zhicheng Pan [1,2] and Mingwang Pan [1,2,*]

1 Department of Polymer Materials and Engineering, School of Chemical Engineering and Technology, Hebei University of Technology, Tianjin 300130, China
2 Hebei Key Laboratory of Functional Polymers, Hebei University of Technology, Tianjin 300130, China
* Correspondence: mwpan@126.com; Tel.: +86-22-6020-2054

3. RESULTS AND DISCUSSION

3.6.2 Influence of Different MFWPU/IBVE Mass Feed Ratio on Scratch Self-Healing Performance of the Composite Coatings

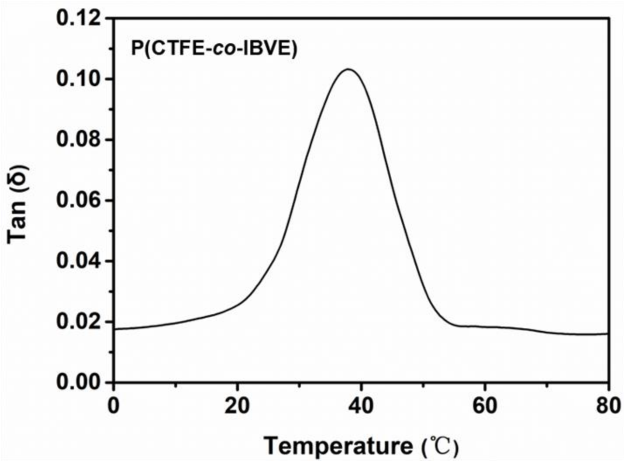

Figure S1. DMA spectra of P(CTFE-*co*-IBVE).

3.7 UV Resistance and Adhesion Performance of Composite Coatings

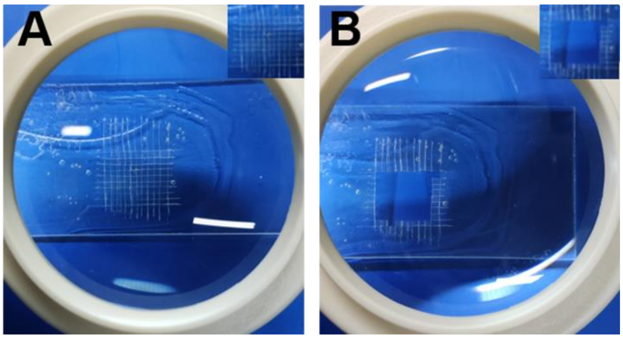

Figure S2. Adhesion performance determination of the P(CTFE-*co*-IBVE) coating by cross-cut test: (A) glass-based coating before 3M tape application; (B) glass-based coating after 3M tape application.

In order to test adhesion performance of the P(CTFE-*co*-IBVE) coating and P(CTFE-*co*-IBVE)/MFWPU composite coating prepared with mass feed ratio of MFWPU and IBVE being 1:5 on glass or tinplate substrates, 100 grids are firstly cut

out with a cutting tool. Subsequently, 3M adhesive tape is pasted on these small grids and removed quickly. The peeling state of P(CTFE-*co*-IBVE) coating and P(CTFE-*co*-IBVE)/MFWPU composite coating on the small grids are displayed in Figure S2 and Figure 11B, respectively. It can be clearly seen that most of the P(CTFE-*co*-IBVE) coatings (> 65%) on the grids are peeled off while the joint of the incision in P(CTFE-*co*-IBVE)/MFWPU composite coating is completely flat and the coating within each grid does not be peeled off. Standard ASTM D3359 describes a standard method for quantifying the adhesion degree of protective coatings on substrates using grades 5B, 4B, 3B, 2B, 1B and 0B respectively, among which grade 5B has the highest adhesion to the substrate while 0B has the lowest adhesion. The adhesion grade of the two kinds of prepared coatings can be judged in terms of the standard.
